# Acute Onset Collagenous Colitis with Unique Endoscopic Findings

**DOI:** 10.1155/2014/986092

**Published:** 2014-12-25

**Authors:** Rintaro Moroi, Katsuya Endo, Masatake Kuhroha, Hisashi Shiga, Yoichi Kakuta, Yoshitaka Kinouchi, Tooru Shimosegawa

**Affiliations:** Division of Gastroenterology, Department of Internal Medicine, Tohoku University Graduate School of Medicine, 1-1 Seiryo, Aoba-ku, Sendai 980-8574, Japan

## Abstract

We experienced a rare case of 72-year-old woman with acute onset collagenous colitis (CC) induced by lansoprazole. The patient developed acute abdominal pain, watery diarrhea, and melena that are quite rare in usual CC. We could find the characteristic colonoscopic findings such as active long liner ulcers in the patient. We also observed the healing courses of these unique findings. Our case indicates two important points of view. (1) CC sometimes develops with acute onset symptoms which resemble those of ischemic colitis. (2) Colonoscopy would be useful and necessary to distinguish acute onset CC and ischemic colitis.

## 1. Introduction

Microscopic colitis (MC) is a disease characterized by chronic, nonbloody watery diarrhea and few or no endoscopic abnormalities [1]. MC comprises two histological subtypes, collagenous colitis (CC) and lymphocytic colitis (LC). Both diseases are characterized by the presence of an inflammatory infiltrate in the lamina propria but differentiated by having either a thickened collagen band in CC or an increased amount of intraepithelial lymphocytes in LC [2]. It is reported that there are much more LC patients in western countries than in Japan. In Japan, there are few LC patients, and CC is much more common [3]. Particularly, the cases of CC induced by lansoprazole are reported to be increasing in Japan [3–8] in recent years.

In the original description, MC has few or no endoscopic abnormalities [9, 10]. However, in recent years, some unique endoscopic findings have been reported in CC patients mainly by Japanese endoscopists. Several case reports described that mucosal abnormalities such as longitudinal ulcer [3, 4, 8, 11, 12], mucosal tears [13, 14], nodular appearance [15], abnormal vascular patterns [10, 16–18], or edema [18] were recognized in CC patients. These findings could be specific for CC suggesting that the original definition of CC in which there are few or no endoscopic abnormalities has to be changed.

CC is originally described as presenting with chronic diarrhea. Actually, most of the CC patients gradually develop chronic continuing watery diarrhea. In CC patients, acute onset symptoms such as acute abdominal pain and melena are thought to be rare. In recent years, a few acute onset cases have been reported [4, 8, 12]. However, the clinical courses and endoscopic findings of acute onset CC are not well understood because of the small number of the patients.

We experienced a rare case of lansoprazole-induced CC with acutely developed abdominal pain and melena. We could also find the characteristic endoscopic findings in this patient and observe the healing courses of these unique findings. In this report, we described the details of clinical courses and endoscopic findings of the patient and made a literature review.

## 2. Case Report

**Figure 1:**
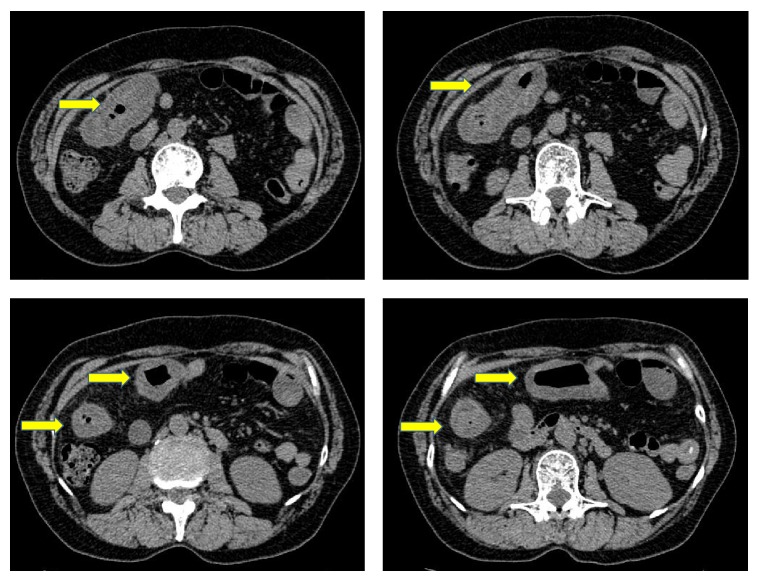
Abdominal computed tomography test revealed the slight edema of the ascending to transverse colon.

**Figure 2:**
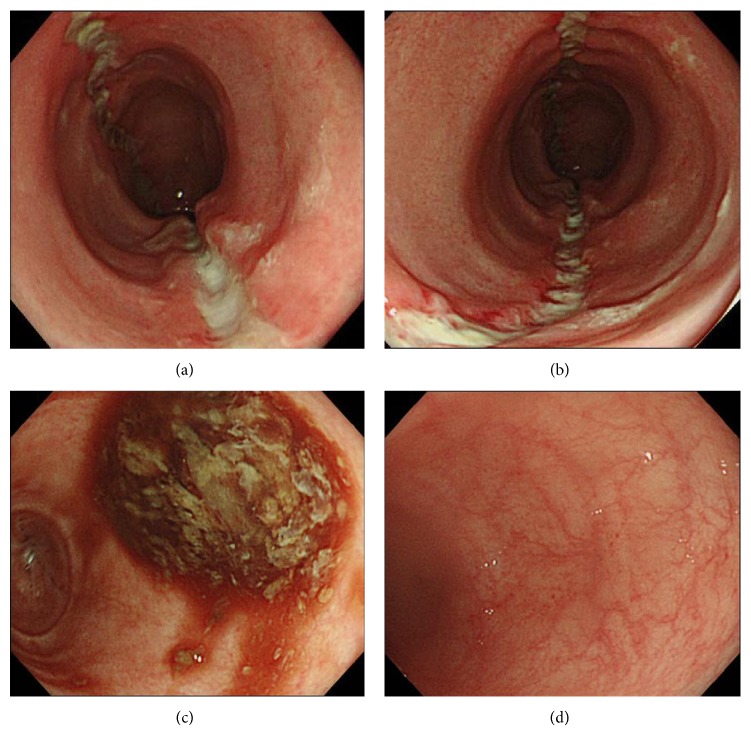
(a, b) Two long linear ulcers were observed in the transverse colon. Neither edema nor reddening was observed around these ulcers. (c, d) From the descending colon to the rectum, the appearances of the mucosa were normal, and the pool of watery-bloody stool was observed.

**Figure 3:**
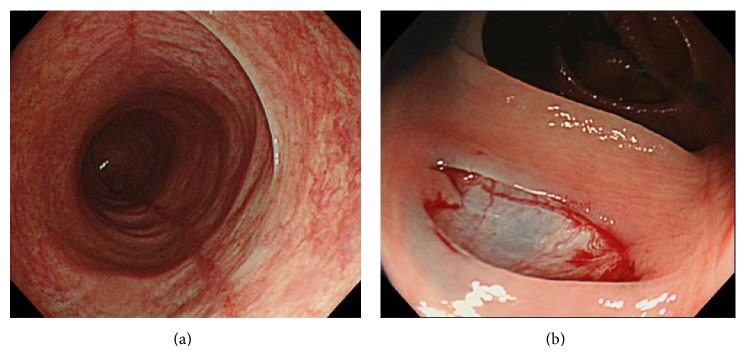
(a) A second colonoscopy was performed two months after the first colonoscopy. The liner ulcers dramatically tended to be healed. (b) Mucosal tear occurred when biopsy specimen was taken from the ascending colon.

**Figure 4:**
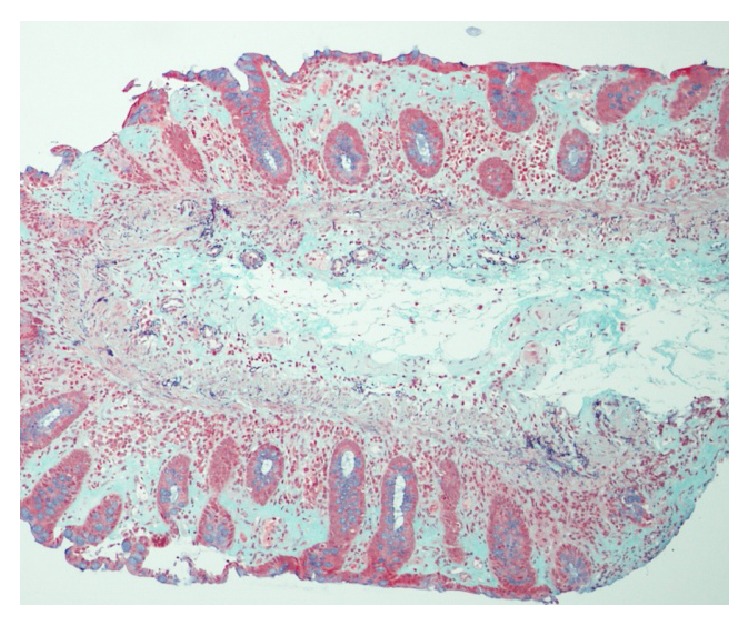
Histological findings of the biopsy specimen revealed the thickened collagen band in the subepithelial layer (Masson trichrome ×100).

**Figure 5:**
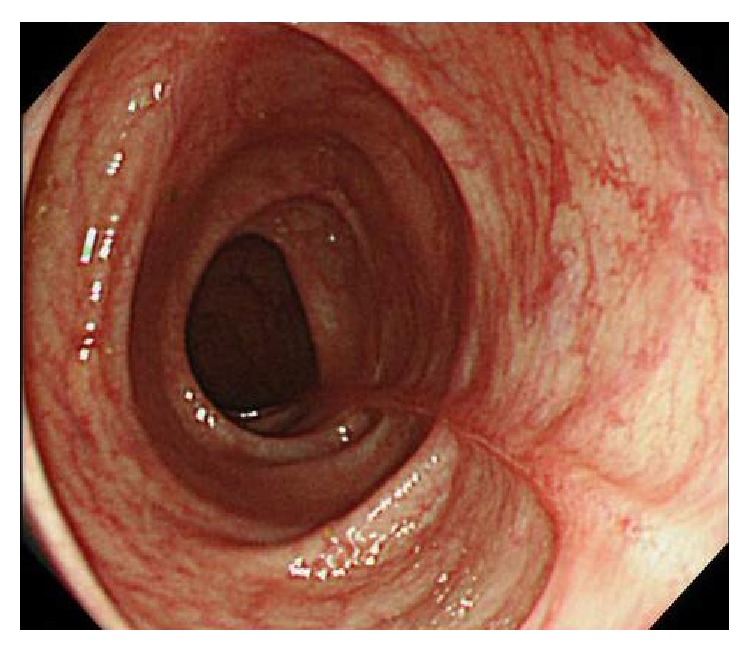
The colonoscopy performed 6 months after stopping lansoprazole showed that liner ulcers were completely healed and became scarred.

**Figure 6:**
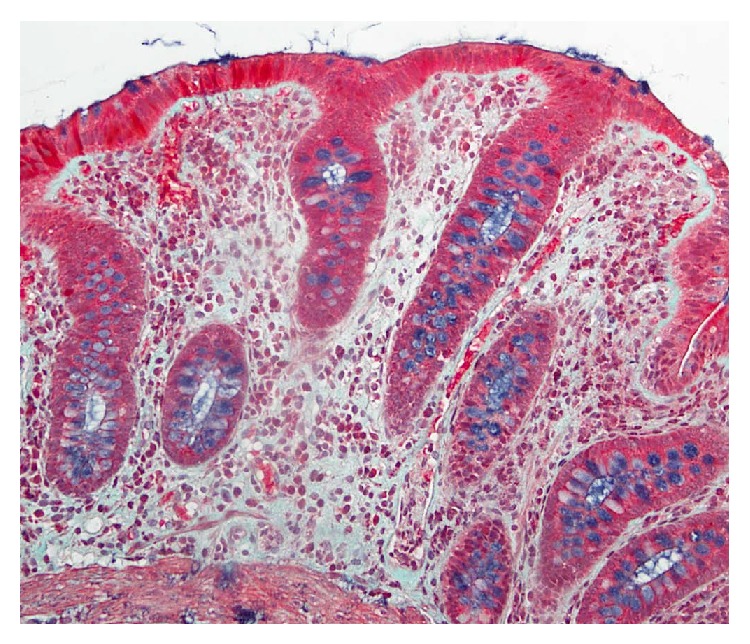
Disappearance of collagen band was confirmed from the histological findings of the ascending colon biopsy specimen (Masson trichrome ×400).

A 72-year-old woman started to take lansoprazole for treatment of the firstly diagnosed reflux esophagitis. At about three months after the first administration of lansoprazole, she suddenly complained of abdominal pain, watery diarrhea, and hematochezia. Therefore, she was admitted to our hospital urgently. Laboratory data revealed white blood cells were 9900/*μ*L; C-reactive protein was 0.9 mg/mL. Other laboratory data are almost within normal range (Table 1). Abdominal computed tomography test (CT) revealed slight edema of the ascending to transverse colon (Figure 1). Because the bloody stool was observed, we performed urgent colonoscopy in order to detect the origin of bleeding. Colonoscopic findings revealed two liner long ulcers in the transverse colon. Neither edema nor reddening was observed around these ulcers (Figures 2(a) and 2(b)). Because of the pain, we could not insert the endoscope into the cecum and the ascending colon. From the descending colon to the rectum, the appearances of the mucosa were normal, and the pool of watery-bloody stool was observed (Figures 2(c) and 2(d)). Although the endoscopic findings were atypical, ischemic colitis was suspected from the clinical symptoms such as acute onset abdominal pain and hematochezia. The patient was conservatively treated with intravenous fluids and was given nothing by mouth. Abdominal pain and bloody diarrhea disappeared in several days. However, watery diarrhea (7~8 times/day) emerged and continued. We followed up the patient as an outpatient. A second colonoscopy was performed two months after the first colonoscopy. Although the liner ulcers dramatically tended to be healed (Figure 3(a)), mucosal tear occurred when biopsy specimen was taken from the ascending colon (Figure 3(b)). Histological findings of the biopsy specimen revealed the thickened collagen band in the subepithelial layer (Figure 4). Therefore, we diagnosed this patient with CC. We stopped administrating lansoprazole because the drug was considered to cause her CC possibly. After stopping the drug, watery diarrhea gradually disappeared in subsequent three months. A third colonoscopy was performed six months after the second colonoscopy. Liner ulcers were completely healed and became scarred (Figure 5). Disappearance of collagen band was also confirmed from the histological findings of the biopsy specimen (Figure 6). Therefore, we concluded her CC was completely healed only by stopping the administration of lansoprazole.

## 3. Discussion

CC is a distinct clinicopathologic entity characterized by chronic watery diarrhea and the subepithelial deposition of collagen band [19]. The disease usually develops gradually with chronic watery diarrhea. Mechanisms of chronic diarrhea in CC have been considered as follows: (1) reduced net absorption of sodium and chloride, (2) some component of active secretion of chloride, and (3) barrier of water reabsorption due to collagen band [20]. In CC patients, acute onset symptoms such as abdominal pain or melena are quite rare. According to the database searches, only 3 cases with acute symptoms have been reported [4, 8, 12] (Table 2). In these 3 cases, acutely developed abdominal pain was observed in all cases, and bloody stool was observed in 2 of 3 cases. All 3 cases had longitudinal ulcer on endoscopic findings. Our case was also a very rare case of CC complaining of acute onset abdominal pain, watery diarrhea, and hematochezia. The present case had unique endoscopic findings which are similar to those in the previous reports. Thus the acute onset CC seems to have common clinical characteristics and endoscopic findings. Here, we have to recognize that clinical symptoms of acute onset CC resemble those of ischemic colitis. When a patient complains of acute abdominal pain with bloody stool, CC should be considered in the differential diagnosis of ischemic colitis. It is important to perform colonoscopy because it could be difficult to distinguish CC from ischemic colitis only by the clinical symptoms.

Endoscopic findings of collagenous colitis are generally thought as no or minimal abnormalities. However, some recent reports have revealed that mucosal abnormalities, such as longitudinal ulcer [3, 4, 8, 11, 12], mucosal tears [13, 14], nodularity, abnormal vascular pattern [10, 16–18], or edema [18], were characteristic endoscopic findings of CC. As for the endoscopic findings of acute onset cases, hemorrhagic, sharply demarcated-longitudinal ulcers were observed [4, 8, 12]. The endoscopic findings of our case also showed active long liner ulcers similar to those of previous reports. In our case, the mucosal tear was also observed when taking the biopsy specimen. Several previous reports assumed that subepithelial collagen deposits cause the mucosal stiffness and friability [13, 21]. The liner ulcers and mucosal tears in our case could possibly be induced by the mucosal fragility caused by the depositions of collagen band. We hypothesized that mucosal stiffness and friability split the membrane sharply in acute phase which led to the acute abdominal pain and hematochezia. According to the previous reports and the experience of our case, active liner longitudinal ulcer would be a specific feature in acute onset CC. Here, we have to focus on the differences between CC and ischemic colitis in endoscopic findings. As previously described, the longitudinal ulcer of acute onset CC resembles that of ischemic colitis. The shortcoming with this case report is that we did not take a biopsy specimen at the first time endoscopy. However, the differences can be observed between two diseases. In common, ischemic colitis presents the longitudinal ulcers accompanied by circumferential mucosal edema [22]. On the other hand, acute onset CC presents sharply demarcated long liner ulcers without any edema or redness. Therefore, it can be possible to distinguish two diseases from these endoscopic findings. We think endoscopic examination might be not only useful, but also necessary to differentiate acute onset CC from ischemic colitis.

In conclusion, we experienced a rare case of lansoprazole-induced collagenous colitis with acutely developed abdominal pain and melena. We could also find the characteristic endoscopic findings in the patient and observe the healing courses of these unique findings. Our case indicates two important points of view. (1) CC sometimes develops with acute onset symptoms such as acute abdominal pain and melena which resemble those of ischemic colitis. (2) Acute onset CC could have characteristic active liner ulcers in the colon. Therefore, colonoscopy would be useful and necessary to distinguish acute onset CC and ischemic colitis.

Although our case and previous reports figured out the characteristics of acute onset CC, additional cases should be needed to understand the disease in detail.

## Figures and Tables

**Table 1:** Laboratory data on admission.

WBC	7300/*µ*L	AST	22 IU/L	PT	105.1%
Seg	81%	ALT	30 IU/L	APTT	26.6 sec
Eosino	1%	ALP	198 IU/L		
Baso	0%	TP	5.4 g/dL	IgG	378 mg/dL
Lymph	9%	Alb	3.4 g/dL	IgA	153 mg/dL
Mono	9%	BUN	11 mg/dL	IgM	109 mg/dL
RBC	491 × 104/*µ*L	Cr	0.9 mg/dL	IgG4	6.5 mg/dL
Hb	15.8 g/dL	Na	142 mEq/L	IgE	85.2 IU/mL
Ht	45.4%	K	3.7 mEq/L	sIL-2R	177 U/mL
Plt	26.4 × 104/*µ*L	Cl	105 mEq/L	ANA	Negative
		CRP	0.1 mg/dL		

WBC: white blood cell, Seg: segmented neutrophils, Eosino: eosinophils, Baso: basophil, Lymph: lymphocytes, Mono: monocytes, RBC: red blood cells, Hb: hemoglobin, Ht: hematocrit, Plt: platelets, AST: aspartate aminotransferase, ALT: alanine aminotransferase, ALP: alkaline phosphatase, TP: total protein, Alb: albumin, BUN: blood urea nitrogen, Cr: creatinine, Na: sodium, K: potassium, Cl: chloride, CRP: C-reactive protein, PT: prothrombin time, APTT: activated partial thromboplastin time, IgG: immunoglobulin G, IgA: immunoglobulin A, IgM: immunoglobulin M, IgE: immunoglobulin E, IgG4: immunoglobulin G4, sIL-2R: soluble interleukin-2 receptor, and ANA: antinuclear antigen.

**Table 2:** The list of case reports developing with acute abdomen.

Case number	Year	Symptoms	Colonoscopic findings	Cause (duration)	Treatment	Treatment responses
1	Yusuke et al., 2009 [4]	Abdominal pain, hematochezia, diarrhea	Liner ulcers (S/C)	LPZ induced (2 months)	Stopping LPZ	Good

2	Kitagawa et al., 2013 [8]	Abdominal pain, hematochezia	Liner ulcers (D/C, S/C)	LPZ induced (unknown)	Stopping LPZ	Good

3	Takanashi et al., 2010 [12]	Abdominal pain, diarrhea, vomit	Liner ulcers (T/C-S/C)	LPZ induced (1 month)	Stopping LPZ	Good

4	Our case	Abdominal pain, hematochezia, diarrhea	Liner ulcers (T/C)	LPZ induced (3 months)	Stopping LPZ	Good
